# Measuring Speech Intelligibility and Hearing-Aid Benefit Using Everyday Conversational Sentences in Real-World Environments

**DOI:** 10.3389/fnins.2022.789565

**Published:** 2022-03-17

**Authors:** Kelly Miles, Timothy Beechey, Virginia Best, Jörg Buchholz

**Affiliations:** ^1^ECHO Laboratory, Department of Linguistics, Macquarie University, Sydney, NSW, Australia; ^2^Hearing Sciences – Scottish Section, School of Medicine, University of Nottingham, Glasgow, United Kingdom; ^3^Department of Speech, Language and Hearing Sciences, Boston University, Boston, MA, United States

**Keywords:** speech intelligibility, hearing aid benefit, realistic speech, clinical assessment development, speech in noise, ECO-SiN

## Abstract

Laboratory and clinical-based assessments of speech intelligibility must evolve to better predict real-world speech intelligibility. One way of approaching this goal is to develop speech intelligibility tasks that are more representative of everyday speech communication outside the laboratory. Here, we evaluate speech intelligibility using both a standard sentence recall task based on clear, read speech (BKB sentences), and a sentence recall task consisting of spontaneously produced speech excised from conversations which took place in realistic background noises (ECO-SiN sentences). The sentences were embedded at natural speaking levels in six realistic background noises that differed in their overall level, which resulted in a range of fixed signal-to-noise ratios. Ten young, normal hearing participants took part in the study, along with 20 older participants with a range of levels of hearing loss who were tested with and without hearing-aid amplification. We found that scores were driven by hearing loss and the characteristics of the background noise, as expected, but also strongly by the speech materials. Scores obtained with the more realistic sentences were generally lower than those obtained with the standard sentences, which reduced ceiling effects for the majority of environments/listeners (but introduced floor effects in some cases). Because ceiling and floor effects limit the potential for observing changes in performance, benefits of amplification were highly dependent on the speech materials for a given background noise and participant group. Overall, the more realistic speech task offered a better dynamic range for capturing individual performance and hearing-aid benefit across the range of real-world environments we examined.

## Introduction

Among the primary functions of speech-in-noise testing are the prediction of speech intelligibility and device benefit outside the clinic or laboratory conditions in which testing is conducted. However, numerous studies have identified discrepancies between the results of speech testing and self-reported speech understanding and device benefit in everyday settings (Working Group on Speech Understanding, Committee on Hearing, Bioacoustics, and Biomechanics, 1988; Cord et al., 2004; Walden and Walden, 2004; Pronk et al., 2018; Wu et al., 2019). For example, using the Hearing in Noise Test (HINT; Nilsson et al., 1994), Cord et al. (2004) found that benefit from directional microphones measured in the laboratory was not predictive of perceived benefit outside the laboratory. Using the same speech test, Wu et al. (2019) found benefits of directional microphones and digital noise reduction but found no such benefits using self-report scales. Similarly, Walden and Walden (2004) found a lack of evidence for any relationship between aided or unaided QuickSIN (Killion et al., 1998) results and subjective ratings of hearing aid benefit once age was taken into account. Speech tests appear to be particularly prone to overestimating real-world outcomes, often showing overly high word recognition scores at rather low (negative) signal-to-noise (SNR) ratios. Such over-estimation is problematic because it can mask the need for further rehabilitation or device optimization and can also disguise rehabilitation and device benefits through ceiling effects. That is, overestimation of speech intelligibility can both underplay and overplay the benefit of interventions. A related problem arises when measuring the speech reception threshold (SRT), in which the SNR is adapted to reach a certain performance point (e.g., 50% correct word identification). Even though the SRT is widely used in clinics, as it is quick and avoids floor and ceiling effects, it results in rather arbitrary test SNRs that are driven by the listener’s performance rather than by real-world SNRs.

Overestimation of real-world performance has led researchers to identify the need for more challenging speech tests (Wackym et al., 2007; Gifford et al., 2008). However, common strategies that may be used to increase the difficulty of speech tests tend to result in speech test materials that are less, rather than more, representative of everyday speech signals. For example, testing at highly negative SNRs increases test difficulty but does not reflect conditions in which people usually need to understand speech, or conditions to which hearing aid features such as compression or adaptive beamforming are best suited or are most likely to be in operation. Word or syllable recognition tasks are more challenging than sentence tests (see for example Olsen et al., 1997) but do not provide the many levels of context normally available to the listener. And, speech tests that are paired with concurrent tasks, such as memorization, are more challenging than singleton tasks but do not closely reflect the cognitive load of everyday speech perception, such as procedural memory demands (Caplan, 2016). It is therefore unlikely that making speech tests more difficult in ways that serve to make speech materials less similar to natural speech signals will provide greater external validity or more accurate real-world predictions.

To create speech tests which can provide more generalizable results it is necessary to account for the cause of overestimation of real-world performance, rather than finding arbitrary ways to make speech tests more challenging. A potential cause can be seen if we consider the differences in perceptual cues provided to listeners by clear speech of the type employed in speech test materials, and conversational speech that is frequently encountered in daily life. Like any complex signal originating in the environment, speech signals consist of multiple redundant cues (Brunswik, 1955). These cues are in a probabilistic, rather than a deterministic, relationship with perceptual targets such as articulated speech features or segments (Blumstein and Stevens, 1981; Heald et al., 2016). Speech tests may overestimate real-world speech perception abilities because speech test materials provide much more robust or reliable segmental cues than are available in conversational speech (Payton et al., 1994; Ferguson and Kewley-Port, 2002; Ferguson, 2012; Ferguson and Quene, 2014). In contrast to clear speech, spontaneous, conversational speech is characterized by high rates of phonetic reduction (Johnson, 2004; Ernestus et al., 2015; Tucker and Ernestus, 2016) and relatively high and variable articulation rates (Miller et al., 1984). For example, excised portions of conversational speech are often unintelligible in isolation (Pollack and Pickett, 1963; Winitz and LaRiviere, 1979), indicating that to understand conversational speech, listeners cannot rely on segmental cues to the extent possible when listening to clear speech. As a result, clear speech of the type employed in speech test materials is more intelligible than conversational speech (Krause and Braida, 2004) but less representative.

By this logic, one approach to improving the predictive capabilities of speech testing is to incorporate features of conversational speech, such as phonetic reductions and realistic speech rates, into the test materials. Including features found in conversational speech has the dual benefit of increasing both the difficulty and realism of speech tests. We recently took this approach in developing the Everyday COnversational Sentences in Noise (ECO-SiN) test (Miles et al., 2020). The ECO-SiN materials were derived from interlocutors conversing in different kinds of realistic background noise, presented via open headphones. This naturally led to variations in vocal effort (e.g., Lombard speech; Lombard, 1911) as well as other accommodations in speaking rate and style (Cooke et al., 2014; Beechey et al., 2018). As a result, when ECO-SiN speech is presented in the noise in which it was produced, it sounds natural and avoids mismatches in level and spectra that listeners are sensitive to Hendrikse et al. (2019).

Our expectation is that the naturalistic aspects inherent to the ECO-SiN sentences will make them less intelligible than clearly articulated sentences typical of existing speech tests. However, at the same time, their vocal effort is appropriate for situations involving background noise, which should enhance the SNR at mid to high frequencies (Badajoz-Davila and Buchholz, 2021). The potential speech intelligibility benefit provided by this SNR boost may interact with the hearing status of the listener if hearing loss restricts access to the additional speech information due to limited audibility, temporal fine structure processing, or spatial processing (e.g., Rana and Buchholz, 2018). It is unclear how the combined effect of these different aspects of realistic effortful speech will affect intelligibility, particularly in realistic noise, and how this may interact with hearing loss and non-linear amplification provided by hearing aids.

To better understand the effect of using more realistic speech materials on hearing outcomes, we directly compared the intelligibility of the highly realistic ECO-SiN sentences to that of more traditional sentences when each were presented in six different realistic background noises. The speech and noise signals were presented at their realistic (fixed) levels (and thus SNRs) and performance was quantified by the percentage of words correctly recognized. Our evaluation included young listeners with normal hearing as well as older listeners with hearing loss, who are ultimately the target population for new and more effective approaches to speech testing. Listeners with hearing loss were assessed unaided and aided to also determine the effect of hearing-aid amplification on speech scores. The outcomes of this exploratory study are intended to highlight the advantages (and possible disadvantages) of increasing the realism of the speech materials in the assessment of speech perception in realistic background noise.

## Materials and Methods

### Participants

**FIGURE 1:**
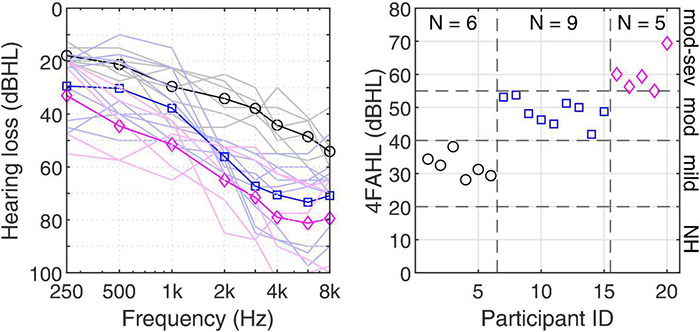
Pure-tone audiograms of the participants with hearing loss averaged across ears (left panel) and their corresponding 4FAHL (right panel). The thin lines in the left panel refer to the individual audiograms and the thick lines with symbols to the audiograms averaged within groups.

Ten young adults with normal hearing (NH) and 20 older adults with hearing loss were recruited as part of a larger study. All participants reported that they were native Australian-English speakers and had no known cognitive or neurological problems. The NH group had audiometric thresholds below 20 dB HL at all audiometric frequencies between 250 and 8,000 Hz. The requisites for admission into the group with hearing loss were symmetrical sensorineural hearing loss with no more than one audiometric pure-tone threshold differing by more than 10 dB between the ears. Four frequency (0.5, 1, 2, and 4 kHz) average hearing loss (4FAHL) was calculated for each individual, and participant groups were established based on the following criterion according to Clark (1981): mild (20 dB HL ≤ 4FAHL < 40 dB HL); moderate (40 dB HL ≤ 4FAHL < 55 dB HL), and moderate-severe (55 dB HL ≤ 4FAHL < 70 dB HL) hearing loss. For those with mild losses, we used the less fine-grained distinction between slight and mild classifications, as per Jerger and Jerger (1980). This grouping was employed as it is how the on-site audiology clinic categorized patients, and as such, how our recruitment efforts were structured. Descriptive statistics of the participants are summarized in Table 1. Using multiple two-sample *t*-tests found no significant differences in age between the three groups with hearing loss (*p* > 0.1) but showed that 4FAHLs were significantly different (*p* < 0.05 using Bonferroni corrections). Figure 1 (left panel) illustrates the individual audiograms (thin lines, averaged across the ears) and the group averages (thick lines) for each of the groups with hearing loss (mild, moderate, and moderate-severe) along with the individual 4FAHLs (right panel). Participants received monetary gratuity for participating in the study. The study was approved by the Macquarie University Human Research Ethics Committee.

**TABLE 1:** Descriptive statistics of the 10 NH participants and 20 participants with hearing loss.

	NH participants	Participants with hearing loss			
		All	Mild	Moderate	Moderate-severe
Number	10	20	6	9	5
Age (Years)	23.1 ± 4.7	74.2 ± 5.2	74.2 ± 4.2	71.6 ± 5.2	76.8 ± 5.2
4FAHL (dB HL)	< 20	47.0 ± 11.4	32.3 ± 3.6	48.7 ± 3.9	60.0 ± 5.6

### Sentence Materials

The realistic sentence materials were drawn from the ECO-SiN corpus (cf. Miles et al., 2020). The ECO-SiN corpus comprises 192 naturally spoken sentences, in which four lists of 16 sentences were spoken with three different vocal efforts. The average sentence length is 6.3 words, and an example sentence is “That discovery was like really interesting for me.” In brief, the sentences were extracted from two people engaging in unscripted conversation while they listened to three different realistic background noises from the ARTE database (Weisser et al., 2019b); a church, an indoor café, a busy food court (see Table 2) via highly open headphones. The background noises were selected based on the conversational speech levels determined by Weisser and Buchholz (2019). The resultant speech levels corresponded to normal, raised, and loud vocal efforts as described in ANSI-S3.5. (1997). All ECO-SiN sentences presented here were spoken by one Australian-English speaking female talker. The female talker was chosen (as opposed to the other male talker of the ECO-SiN corpus) to provide the best point of comparison with the reference sentences (see below) which are spoken by a female talker.

**TABLE 2:** Details of the realistic environments and speech materials.

ID	Environment	Noise level	RT (Sec)	Speech level (dB SPL)	SNR (dB)	Vocal effort	
		(dB SPL)				ECO-SiN	BKB
1	Office	58	0.2	63.4	5.4	Normal	N/A
2	Church	62.5	1.2	65.4	2.9		
3	Living room	66.9	0.2	67.4	0.4	Raised	
4	Cafe	71.4	1.1	69.3	−2.1		
5	Dinner party	75.9	0.4	71.3	−4.6	Loud	
6	Food court	80.3	1	73.3	−7.1		

*Numbers are rounded.*

The more traditional (reference) materials were drawn from a corpus of “BKB-like” sentences created by the Cooperative Research Centre for Cochlear implant and Hearing Aid Innovation (CRC HEAR). These sentences are similar to the original BKB sentences (Bench et al., 1979), however, the BKB-like corpus contains more sentences and was recorded with an Australian-English speaking female. The corpus has 80 lists in total, with each list consisting of 16 sentences. The average sentence length is 4.9 words and a n example sentence is “The clown had a funny face.” The scripted and clearly spoken sentences were produced in a sound-attenuated booth with the intention of being easily understood by 5-year-old children. The average spectrum of the BKB-like sentences is normalized to match the long-term average speech spectrum (LTASS) described by Byrne et al. (1994). The BKB-like sentences (hereafter referred to as BKB sentences) are widely used in research laboratories (e.g., see Dawson et al., 2013; Rana and Buchholz, 2016; Bentsen et al., 2019) and hearing clinics throughout Australia and were therefore considered here as an appropriate reference material.

**FIGURE 2:**
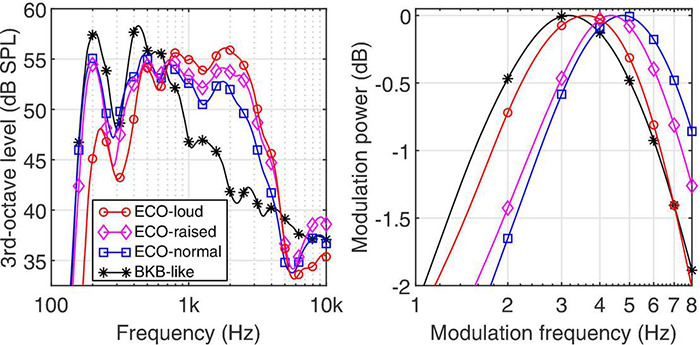
Third-octave spectrum (left panel) and modulation spectrum (right panel) for the different speech materials and effort levels at an average broadband level of 65 dB SPL.

The average spectrum of the speech materials is shown in Figure 2 (left panel) for the BKB sentences (black stars) and the ECO-SiN sentences, separately for the normal (blue squares), raised (magenta diamonds), and loud (red circles) vocal effort. The spectra were derived in 3rd-octave bands for an unweighted RMS level of 65 dB SPL and averaged across all available sentences (i.e., the 1,280 sentences of the BKB material and the 64 sentences for each effort level of the ECO-SiN material). Compared to the BKB sentences, the ECO-SiN sentences provide a significant energy boost at mid-frequencies between 800 and 4,000 Hz, which further increases with increasing vocal effort level.

The corresponding temporal modulation spectra of the different speech materials are shown in the right panel of Figure 2. The modulation spectra were derived by concatenating all sentences for a given speech material into a single signal, which was then bandpass filtered using an A-weighting filter to focus roughly on the frequency range most relevant for speech perception. The amplitude of the resulting signal was squared, analyzed by a modulation filterbank with one-octave wide filters, and the power in each modulation channel calculated in dB. The resulting modulation spectrum was then normalized to its maximum value for easier comparison across speech materials. The modulation spectra exhibit a modulation bandpass characteristic with a center frequency that changes across speech materials and effort levels. Considering the center frequency as a rough estimator of the average talking rate, the talking rate in the BKB sentences is the slowest (3.2 Hz) and for the ECO-SiN sentences decreases with increasing vocal effort: normal (4.8 Hz), raised (4.4 Hz), and loud (3.6 Hz).

### Acoustic Environments

The background noises were drawn from the ARTE database (Weisser et al., 2019b), which were recorded with a 62-channel hard-sphere microphone array and encoded into the higher-order Ambisonics (HOA) format. They were then decoded here for simulated playback with the spherical 41-channel loudspeaker array inside the anechoic chamber of the Australian Hearing Hub, Macquarie University. Table 2 shows the selected environments, their associated noise levels (i.e., the unweighted sound pressure level calculated over the entire recording of 150 s) and reverberation times (RT) in free-field, and the mapping of the ECO-SiN and BKB speech materials to the environments. The environments consisted of (1) an open plan office that was separated into cubicles using acoustically absorptive wall dividers, and contained people typing, chatting, and talking on the phone; (2) a small church with people entering and chatting before service; (3) a small living room with access to a kitchen in the back, with a television presenting commercials and kitchen sounds from the back; (4) an indoor café at medium occupancy with people chatting and diverse kitchen and coffee making noises; (5) a dining room with eight people chatting and laughing over a table and background music; and (6) a very large and noisy food court in a shopping mall at lunch time, which produced a very diffuse and stationary babble-like noise.

The speech levels for the six different environments (see Table 2) were derived from Equation 9 of Weisser and Buchholz (2019), who measured realistic SNRs in different realistic environments, including the ones used in the current study. In this equation, the gender-averaged SNR of two talkers sitting at a head-to-head distance of 1 m was considered, and the noise levels were slightly adjusted from their original levels to result in fixed SNR steps of 2.5 dB. To maximize the realism of the ECO-SiN sentences, and thereby to optimize their perceptual integration with the background noise, realistic room reverberation was added by convolving the individual sentences with multi-channel Room Impulse Responses (RIRs). The RIRs were taken from the ARTE database (Weisser et al., 2019b) and measured in the real-world environments with a loudspeaker at a distance of 1.3 m in front of the 62-channel microphone array. As for the noise recordings, the measured RIRs were encoded into the HOA format and decoded for simulated playback with the 41-channel loudspeaker array. Thereby, to compensate for the difference in the measured (1.3 m) and simulated (1 m) source-receiver distance, the direct sound was separated from the individual RIRs using a frequency-dependent time window, amplified such that the broadband direct-sound-to-reverberation energy ratio in free-field was increased by 20 × log(1.3 m/1 m) = 2.3 dB, and then added back to the RIRs. To reduce the apparent source width of the direct sound, its impulse response was integrated across all 41 loudspeaker channels before it was added back to only the frontal channel of the RIRs. The anechoic BKB sentences were presented only from the frontal position.

Note that the speech levels given in Table 2 refer to the average broadband free-field levels of the anechoic BKB sentences and the direct-sound only (i.e., anechoic) ECO-SiN sentences. The free-field levels of the reverberant ECO-SiN sentences were slightly higher than the values shown in Table 2, the reverberation providing an increase in the effective test SNR by: + 0.8 dB, church: + 0.1 dB, living room: + 1.2 dB, café: + 0.8 dB, dinner party: + 1.6 dB, and food court: + 0.5 dB. For a detailed description of the microphone array recording, HOA encoding and decoding, and the RIR manipulation process see Weisser et al. (2019b).

### Binaural Playback and Hearing-Aid Amplification

The loudspeaker signals for the different noise and speech conditions were transformed into binaural signals by simulating their playback via the 41-channel loudspeaker array to the in-ear microphones of a Bruel and Kjaer (Skodsborg Vej 307, 2850 Naerum, Denmark) type 4128C Head and Torso Simulator (HATS). Additionally, to enable the integration of a pair of hearing aids in the binaural playback, behind-the-ear (BTE) hearing aid satellites were placed above the left and right ear of the HATS. These purpose-built satellites were provided by Sonova AG (Laubisrütistrasse 28, 8712 Stäfa, Switzerland) and included front and rear microphones that were connected to a purpose-built pre-amplifier. The playback simulation path included individual loudspeaker equalization filters as well as measured impulse responses from each of the 41 loudspeakers to the six microphones at the left and right ears of the HATS: two in-ear microphones and four hearing aid microphones. However, only the front hearing aid microphones were used in this study to realize an omni-directional hearing aid input. Further details of the playback simulation process can be found in Weisser and Buchholz (2019).

**FIGURE 3:**
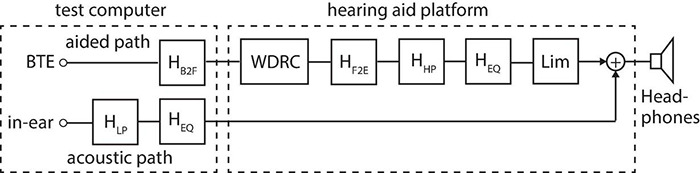
Block diagram of the acoustic and aided signal path from the HATS’ in-ear and BTE (front) microphone to the headphones for binaural playback. Only the pathway for the left or right ear is shown here.

Figure 3 illustrates the implemented acoustic and aided signal path from the in-ear and front BTE microphones to the headphones used for binaural playback in the listening tests. Since the signal paths are identical at the left and right ear only one ear is shown here. The acoustic path describes the sound that arrives directly at the listener’s ear drum (i.e., the in-ear microphone) and circumvents any hearing aid fitting (or ear mold). This path includes a low-pass filter, *H*_*LP*_, to mimic the passive attenuation of the hearing aid fitting as well as a headphone equalization filter, *H*_*EQ*_. The equalization filter ensured a flat frequency response of the headphones when measured on the HATS. The aided path describes the signal path from the hearing aid microphone via the hearing aid processing to the headphones. This path includes (1) a BTE microphone to free-field transformation filter, *H_*B*2F_*, that removes the acoustic head shadow for a frontal sound source and provides a free-field equivalent output; (2) a multi-channel wide dynamic range compressor (WDRC) as the main hearing aid processing; (3) a free-field to ear-drum transformation filter, *H_*F*2E_*, that basically reintroduces the acoustic head shadow for a frontal sound source but as recorded by the in-ear microphone; (4) a high-pass filter to simulate the limited sensitivity of the hearing aid receiver at low frequencies; (5) the same headphone equalization filter used in the acoustic path; and (6) an instantaneously acting broadband limiter, *Lim*, to protect the listener from excessively loud sounds.

A standard desktop computer was used to run the listening tests and to play the different 4-channel speech and noise stimuli via a RME Fireface UC (Audio AG, Am Pfanderling 60, 85778 Haimhausen, Germany) USB sound card to a second desktop computer with an RME Audio Fireface UFX USB sound card. The second computer ran a real-time hearing-aid research platform developed at the National Acoustic Laboratories, Hearing Australia, and presented the (aided) binaural stimuli to the participants via Beyerdynamic (Theresienstrasse 8, 74072 Heilbronn, Germany) DT990 headphones. All stimulus playback was realized at a sampling frequency of 44.1 kHz except for the hearing aid platform, which operated at a sampling frequency of 24 kHz and was band-limited to about 10 kHz.

The low-pass filter, *H*_*LP*_, and high-pass filter, *H*_*HP*_, shown in Figure 3 were both realized by second order Butterworth IIR filters with different cut-off frequencies to approximate the acoustic attenuation by an ear mold with a vent size of 1, 2, and 3.5 mm. The cut-off frequencies were 620, 883, and 1,371 Hz for the low-pass filter and 311, 470, and 926 Hz for the high-pass filter. The filters approximated the gain data provided by Dillon (2001, page 127, Figure 5.11) and Dillon (2001, p. 127, Table 5.1), respectively, and presented a wide range of fittings from an almost open fitting (3.5 mm) to an almost closed fitting (1 mm). For each participant with hearing loss, the vent size was selected based on their low-frequency hearing loss (LFHL) as given by their ear-averaged pure-tone threshold at 500 Hz. Based on a discussion with local audiologists, the vent sizes were 3.5 mm for LFHL ≤ 20 dBHL, 2 mm for 20 dBHL < LFHL ≤ 30 dBHL, and 1 mm for LFHL > 30 dBHL. The WDRC realized basic syllabic compression within 16 independent frequency channels and acted independently across ears. It was fitted to the individual participant (and ear) using the NAL-NL2 gain prescription formula (Keidser et al., 2012). The instantaneous broadband limiter, *Lim*, was part of the sound card of the hearing aid platform and was set to an attack time of 0 ms, a release time of 100 ms, a compression ratio of 6, and a knee-point of 95 dB SPL. The limiter was significantly engaged only for the participants with moderate and moderate-severe losses, and then only in the loudest environments. For NH participants as well as participants with hearing loss in the unaided conditions, materials were presented through the acoustic path only, with the lowpass filter removed (i.e., set to a flat gain of 0 dB; see Figure 3). This rather complicated approach of using headphone reproduction with a hearing aid research platform was chosen here over a multi-loudspeaker system with off-the-shelf hearing aids to maximize control of the entire signal path from the acoustic free field through the hearing aid processing to the signals at the listener’s ears. Arguably, such a system may also be easier to use within a hearing clinic.

### Procedure

Individual word recall ability was measured in the six realistic acoustic environments using both the realistic ECO-SiN and the more traditional BKB sentence materials at realistic (fixed) noise and speech levels, and thus SNRs (see Table 2). The sentences were always presented from the front. The NH participants were tested unaided, and the participants with hearing loss were tested both unaided and aided. Participants were seated together with the test administrator in a sound attenuating test booth with double walls. In each test condition, a 2.3-min-long noise sample was played in a loop and the 16 sentences in a list were presented in random order. Each time a sentence was presented, the participants recalled aloud all the words they heard. The administrator then scored the number of correctly recalled words on a graphical user interface that was invisible to the participant, and a new sentence was played. Preceding each sentence presentation was a 1 kHz beep to signal to the participant that a sentence was about to be played.

The order of the six background noises and the two speech materials (i.e., 12 test conditions) was randomized. These test conditions were blocked for the participants with hearing loss within the unaided and aided conditions due to the required manual reconfiguration of the hearing-aid platform. The two blocks were tested in random order.

## Results

### Speech Intelligibility Scores

**FIGURE 4:**
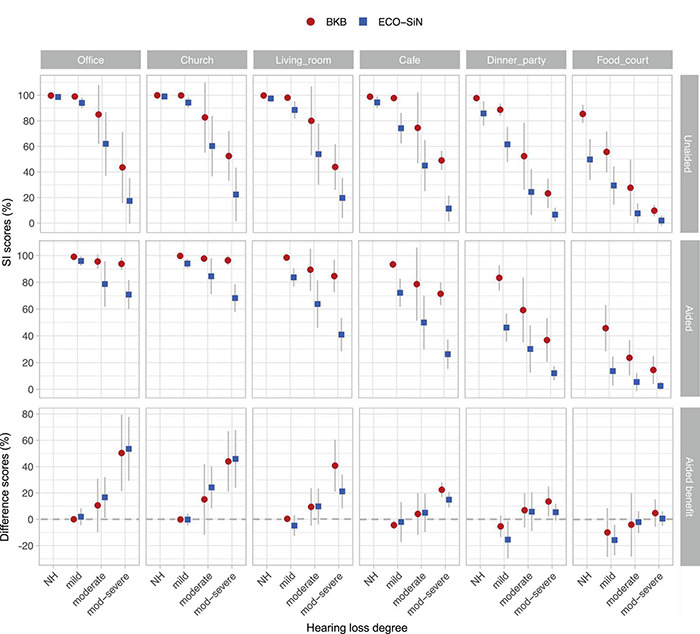
Speech intelligibility (SI) scores obtained with BKB and ECO-SiN sentences. Shown are group means in percent correct (error bars show standard deviations). Top row: unaided listening; middle row: aided listening; bottom row: hearing-aid benefit (difference between aided and unaided scores). Note the different *y*-axis scales.

Figure 4 shows mean intelligibility scores in each environment for unaided (top row) and aided (middle row) listening. Within each panel, data are shown for each listener group and for the two speech materials. For NH listeners the intelligibility scores in the quieter environments were all at ceiling and only decreased in the loudest environments. This decrease was more pronounced for the ECO-SiN than the BKB material, leading to generally higher BKB scores in the louder environments. When listening unaided, all of the participant groups with hearing loss showed higher BKB scores than ECO-SiN scores in all of the environments, but the magnitude of the difference varied with the environment. For listeners with mild loss, the difference increased in the louder environments as the influence of ceiling effects was reduced. For listeners with moderate-severe hearing loss, the opposite pattern was observed, with the difference between BKB and ECO-SiN scores decreasing in the louder environments as floor effects came into play. When amplification was provided for listeners with hearing loss, intelligibility scores generally improved. As for the unaided condition, BKB scores were generally higher than ECO-SiN scores across all environments. Because of the overall shifts in the intelligibility functions, however, the magnitude of the speech material differences varied differently across environments.

To quantify the effect of speech material (BKB vs. ECO-SiN) on unaided and aided speech intelligibility scores, a Bayesian Beta regression model was fitted (Ferrari and Cribari-Neto, 2004) using the R-INLA package (Rue et al., 2017). Intelligibility scores were modeled as proportions as a function of categorical predictor variables for speech material, hearing loss group, and acoustic environment. A random intercept for individual subjects was included to account for repeated measures. The results of this analysis are provided in Table 3. Focusing on the contrast between BKB and ECO-SiN scores, for NH listeners, predicted mean scores were significantly higher for BKB than for ECO-SiN sentences in the café, dinner party, and food court environments (but not in the office, living room, or church environments). For listeners with hearing loss, the difference between speech materials was significant in all environments for both unaided and aided conditions.

**TABLE 3:** Results of the statistical analysis comparing intelligibility scores for the two types of speech materials.

	Degree of HL	Aiding	Difference	Low 95% CI	Upper 95% CI
Office	NH	Unaided	0.55	–1.10	2.44
Office	Mild	Aided	6.17*	0.84	13.77
Office	Mild	Unaided	5.58*	0.61	12.96
Office	Moderate	Aided	17.63*	8.13	29.73
Office	Moderate	Unaided	33.17*	17.39	49.09
Office	Moderate-severe	Aided	24.16*	7.32	43.66
Office	Moderate-severe	Unaided	33.69*	14.30	54.09
Church	NH	Unaided	0.59	–0.82	2.26
Church	Mild	Aided	5.31*	0.79	11.95
Church	Mild	Unaided	5.72*	1.31	12.52
Church	Moderate	Aided	12.88*	5.57	22.92
Church	Moderate	Unaided	28.19*	12.12	44.74
Church	Moderate-severe	Aided	33.14*	16.38	52.30
Church	Moderate-severe	Unaided	35.98*	13.26	57.58
Living room	NH	Unaided	1.54	–0.55	4.22
Living room	Mild	Aided	14.29*	5.17	26.89
Living room	Mild	Unaided	6.71*	0.32	15.51
Living room	Moderate	Aided	25.33*	11.66	40.69
Living room	Moderate	Unaided	28.17*	11.90	44.72
Living room	Moderate-severe	Aided	43.84*	21.74	63.85
Living room	Moderate-severe	Unaided	24.57*	2.01	46.80
Cafe	NH	Unaided	3.77*	0.22	8.49
Cafe	Mild	Aided	18.36*	3.99	34.99
Cafe	Mild	Unaided	18.13*	7.29	32.37
Cafe	Moderate	Aided	26.37*	8.75	43.65
Cafe	Moderate	Unaided	31.01*	12.22	48.55
Cafe	Moderate-severe	Aided	43.28*	18.28	63.98
Cafe	Moderate-severe	Unaided	35.87*	13.83	57.20
Dinner Party	NH	Unaided	6.19*	0.49	13.16
Dinner Party	Mild	Aided	35.22*	14.32	55.21
Dinner Party	Mild	Unaided	19.05*	1.96	37.72
Dinner Party	Moderate	Aided	24.54*	4.31	43.34
Dinner Party	Moderate	Unaided	25.05*	7.26	42.33
Dinner Party	Moderate-severe	Aided	22.36*	2.37	43.46
Dinner Party	Moderate-severe	Unaided	17.60*	3.94	34.55
Food court	NH	Unaided	24.16*	8.98	39.43
Food court	Mild	Aided	26.89*	6.11	47.14
Food court	Mild	Unaided	21.07	–3.04	43.89
Food court	Moderate	Aided	14.67*	6.38	25.18
Food court	Moderate	Unaided	16.89*	7.81	28.26
Food court	Moderate-severe	Aided	5.56	–1.69	15.25
Food court	Moderate-severe	Unaided	8.18*	1.66	18.29

*Significant differences at the p < 0.05 level are indicated with an asterisk.*

### Hearing-Aid Benefit

Hearing-aid benefit was calculated by subtracting the unaided speech intelligibility percentage score from the aided speech intelligibility percentage score for each individual, separately for the BKB and ECO-SiN materials, with positive values indicating that amplification provided an improvement in speech intelligibility. Mean benefits are shown in the bottom row of Figure 4.

Given the complex behavior of the unaided and aided scores described in section “Speech Intelligibility Scores,” the differences between them were also complex and were strongly affected by floor and ceiling effects. The largest aided benefits were observed for the listeners with moderate-severe hearing loss in the quietest environments. In those same environments, ceiling performance tended to reduce or eliminate the measurable benefit for better-performing listeners with milder losses. For the louder environments (e.g., the food court), floor effects meant that benefits of amplification were generally not observed for the listeners with moderate-severe hearing losses. In these louder environments though, better performing listeners who were not at floor demonstrated negative benefits (or “disbenefits”). In some cases, the magnitude of the benefit clearly depended on the type of speech material used.

To quantify the effect of speech material (BKB vs. ECO-SiN) on hearing-aid benefit, a robust regression model with a Student-T noise distribution was fitted to model hearing-aid benefit data which is not constrained to the [0, 1] interval. The results of this analysis are provided in Table 4. Focusing again on the differences between BKB and ECO-SiN materials, this analysis found significantly larger ECO-SiN benefits in the office and church environments for the listeners with moderate hearing loss only. In the living room and café environments, benefits were significantly larger for the BKB materials in the listeners with moderate-severe hearing loss. In the dinner party environment, the effect of speech material was significant only for the listeners with mild hearing loss, who showed larger *disbenefits* for the ECO-SiN materials.

**TABLE 4:** Results of the statistical analysis of the hearing-aid benefits.

Speech	Noise	Degree of HL	Mean	0.025 quant	0.975 quant
BKB	Office	Mild	0.458	–5.934	7.004
ECO-SiN	Office	Mild	–0.357	–7.651	7.386
BKB	Office	Moderate	5.297	–1.436	12.551
ECO-SiN	Office	Moderate	18.321	7.825	28.488
BKB	Office	Moderate-severe	45.190	28.966	60.202
ECO-SiN	Office	Moderate-severe	47.299	34.756	61.061
BKB	Church	Mild	–0.158	–7.207	6.870
ECO-SiN	Church	Mild	0.226	–7.488	7.926
BKB	Church	Moderate	5.536	–1.172	12.925
ECO-SiN	Church	Moderate	22.083	14.138	30.298
BKB	Church	Moderate-severe	43.211	28.015	57.273
ECO-SiN	Church	Moderate-severe	49.562	38.695	60.268
BKB	Living room	Mild	0.452	–6.597	7.538
ECO-SiN	Living room	Mild	–7.927	–15.391	–0.464
BKB	Living room	Moderate	6.428	–1.564	15.105
ECO-SiN	Living room	Moderate	8.907	–1.146	18.075
BKB	Living room	Moderate-severe	39.235	26.273	51.370
ECO-SiN	Living room	Moderate-severe	22.024	11.707	31.259
BKB	Cafe	Mild	–4.199	–11.397	2.991
ECO-SiN	Cafe	Mild	–7.460	–16.114	1.707
BKB	Cafe	Moderate	5.827	–1.727	13.547
ECO-SiN	Cafe	Moderate	6.181	–1.939	14.478
BKB	Cafe	Moderate-severe	22.463	13.925	31.043
ECO-SiN	Cafe	Moderate-severe	13.522	4.797	22.182
BKB	Dinner party	Mild	–5.410	–14.003	2.685
ECO-SiN	Dinner party	Mild	–22.513	–32.531	–11.524
BKB	Dinner party	Moderate	5.629	–2.129	13.866
ECO-SiN	Dinner party	Moderate	6.339	–2.037	15.016
BKB	Dinner party	Moderate-severe	16.276	6.652	25.044
ECO-SiN	Dinner party	Moderate-severe	6.113	–2.016	14.251
BKB	Food court	Mild	–14.831	–23.760	–5.193
ECO-SiN	Food court	Mild	–17.038	–27.093	–7.317
BKB	Food court	Moderate	–0.918	–9.081	8.115
ECO-SiN	Food court	Moderate	–0.622	–7.273	6.120
BKB	Food court	Moderate-severe	6.482	–3.485	15.887
ECO-SiN	Food court	Moderate-severe	0.314	–8.616	9.243

### Relationship Between BKB and Everyday Conversational Sentences in Noise Scores and Benefits

**FIGURE 5:**
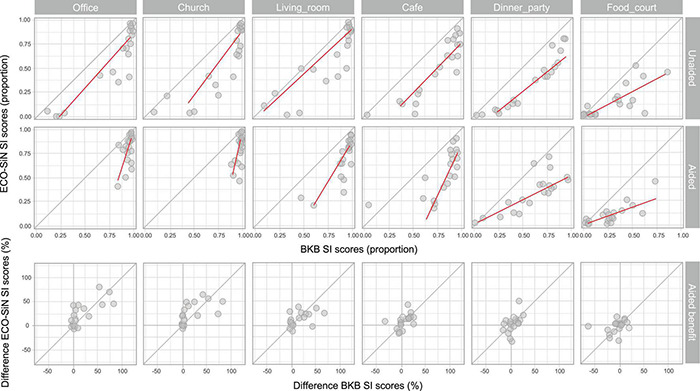
Individual speech intelligibility (SI) scores for ECO-SiN as a function of BKB. Top row: unaided listening; middle row: aided listening; bottom row: hearing-aid benefit (difference between aided and unaided scores).

Figure 5 shows individual listener scores for ECO-SiN sentences as a function of their scores for BKB sentences when listening unaided (top row, excludes NH listeners) and with non-linear amplification (bottom row). Consistent with the observations made in section “Speech Intelligibility Scores,” the majority of the points lie below the diagonal, indicating that ECO-SiN scores were lower than BKB scores achieved by most individuals.

A quantile regression model was fitted to compare the behavior of the individual ECO-SiN scores as a function of BKB scores in each environment and aiding condition with the predicted slopes describing the relative spread of the distributions of ECO-SiN and BKB scores. Quantile regression was used because it is robust to outliers and makes no assumptions about the underlying distribution of the data. The regression lines in Figure 5 show predicted median ECO-SiN score as a function of performance on the BKB task. A slope of 1 would indicate that ECO-SiN scores change at the same rate as BKB scores, whereas a slope greater than 1 indicates that ECO-SiN scores change more than BKB scores and a slope less than 1 indicates that ECO-SiN scores change less than BKB scores. A higher rate of change indicates greater spread of scores and a wider distribution, while a lower rate of change indicates that scores are more concentrated within a small range, corresponding to a narrow distribution such as data accumulating at floor (0, i.e., 0%) or ceiling (1, i.e., 100%).

In unaided conditions, ECO-SiN and BKB scores show very similar spreads in the office (slope = 1.1; CI = 0.97, 1.25; *p* < 0.001), church (slope = 1.28; CI = 0.56, 1.85; *p* < 0.001), living room (slope = 0.93; CI = 0.63, 1.51; *p* < 0.001), and café (slope = 1.06; CI = 0.66, 1.69; *p* < 0.001) environments. In the two loudest environments there is a trend toward lower rates of change in ECO-SiN scores relative to BKB scores, with a slope of 0.75 (CI = 0.48, 1.19; *p* < 0.001) in the dinner party environment and a slope of 0.56 (CI = 0.20, 0.95; *p* = 0.003) in the food court environment. In the aided conditions, a similar trend is seen with slopes becoming progressively shallower in the louder environments. We see high relative rates of change in ECO-SiN scores in the four softest environments including the office (slope = 2.51; CI = 0.20, 3.67; *p* = 0.005), church (slope = 4.32; CI = 0.42 7.31; *p* = 0.014), living room (slope = 1.55; CI = −1.73, 5.28; *p* = 0.39) and café (slope = 1.77; CI = −1.5, 5.56; *p* = 0.33). Very low relative rates of change in ECO-SiN scores occurred in the two loudest environments including the dinner party (slope = 0.46; CI = −2.58, 3.91; *p* = 0.79) and the food court (slope = 0.39; CI = −3.06, 3.83; *p* = 0.84).

A significant relationship can be observed between the individual ECO-SiN and BKB scores for all environments when individuals were unaided, and in the quietest environments when aided. Hence, within many of the individual test conditions, a linear model can reasonably well predict the individual ECO-SiN scores from the corresponding BKB scores. However, this is not the case across the different environments and aiding conditions, where a far more complicated relationship exists between the two speech materials. Hence, knowing a BKB score in a single test condition does not allow prediction of the individual score in another environment nor the benefit provided by non-linear amplification. This is highlighted by the slopes (and distributions) that change drastically across the different test conditions (i.e., across panels in Figure 5) and are insignificant for the louder aided conditions.

Also shown in Figure 5 (bottom row) is the hearing-aid benefit measured using ECO-SiN sentences plotted as a function of the equivalent benefit measured using BKB sentences. This display illustrates the fact that when performance scores are at or near ceiling there is reduced scope to detect performance improvements. Visual inspection of the scatter plots reveals clustering of data around zero on the BKB benefit scale (*x*-axis) in the three softest environments: the office, church, and living room. Clustering around zero on the *x*-axis was less clear in the café and dinner party environments. In the loudest environment, the food court, there was instead evidence of clustering of data around zero on the ECO-SiN benefit scale (*y*-axis).

## Discussion

### Summary and Implications of Results

In this study we demonstrated that by using sentences embedded in a range of real-world environments, with their natural SNRs, the overall difficulty of a speech-in-noise test can be varied in a meaningful way. This means that by selecting the right environment a useful operating point (where scores are away from both ceiling and floor) can be found for listeners across a wide range of hearing abilities. Depending on the specific purpose, the test environment may be selected based on the individual’s hearing loss, their reported speech-in-noise problem, or the relevance of a test environment (e.g., see Mansour et al., 2021). Furthermore, we demonstrated that, within our framework, the choice of speech materials not only affected the realism of the stimuli but also changed the difficulty of the listening task. Specifically, we found that highly realistic sentences from the ECO-SiN corpus resulted in lower speech intelligibility scores overall, as compared to the clearly spoken BKB sentences. We note that this result is broadly consistent with the results of a number of studies that have demonstrated that clear speech is more intelligible than conversational speech in noise for both NH and listeners with hearing loss (Picheny et al., 1985, 1989; Payton et al., 1994; Uchanski et al., 1996; Krause and Braida, 2004, 2009; Krause and Panagiotopoulos, 2019). We also found that while BKB scores were able to reasonably well predict ECO-SiN scores within a given test condition (e.g., regression lines in Figure 5), this linear relationship was weaker in the aided conditions in the louder background noises. In addition to this, the relationship between the different speech materials and the aiding conditions demonstrated the complexity of predicting one score from another when making comparisons across the different environments.

This ability to vary the operating point within real-world speech testing (by selecting the right environment) has important consequences if the aim is to examine the effect of a particular intervention. In our study, this point was made for the case of non-linear hearing-aid amplification. Because intelligibility scores varied substantially across environments, degree of hearing loss, and speech material, so too did the ability to measure a benefit of amplification. For instance, as shown in Figure 4, there was no aided benefit in the office and church environment (for either kind of speech material) for listeners with mild hearing loss. This was because the unaided and aided scores were all at ceiling. Similarly, there was no aided benefit for the listeners with moderate and moderate-severe hearing loss in the food court environment (for either kind of speech material) because both sets of scores were at or near floor. These two examples highlight there are limits on how much benefit/disbenefit (operationalized as the increase or decrease in words correctly understood) that can be measured for a given listener group in a given environment (or SNR). On top of this, we saw an impact of the chosen speech materials on speech scores and hence on hearing-aid benefits. For example, Figure 5 (bottom left) shows that hearing-aid benefits clustered around zero for the BKB sentences in the quieter listening environments, while benefits were observable with ECO-SiN sentences. To summarize, hearing-aid benefit depends heavily on both the environment and on the speech materials used. If the goal is to understand how much a particular listener will benefit from amplification in a particular environment (or range of environments), then we argue that the ECO-SiN test at realistic SNRs provides the most meaningful estimate.

Within the constraints of our measurement approach, two main observations could be made regarding hearing-aid benefit. First, the aided benefit was largest for the listeners with the most severe hearing loss in the quietest conditions. The listeners showed the lowest unaided intelligibility scores in these conditions and thus, had also the largest opportunity to receive a benefit from hearing-aid amplification. This observation is in agreement with previous studies showing greater aided benefit with greater hearing loss (McArdle et al., 2012; Woods et al., 2015) and greater aided benefit when sentences were presented in quiet compared to noise (Mendel, 2007). In addition, it is very likely that their intelligibility scores were limited by reduced audibility, which is the main aspect of hearing loss that can be compensated by hearing-aid amplification. A second observation is that *negative* benefits were observed for the listeners with mild hearing loss in the louder environments. In these conditions, where the overall SNR is negative, speech audibility is not expected to play a significant role because the main limitation is the presence of the noise. Accordingly, it is unsurprising that amplification did not provide any strong improvement in intelligibility. Moreover, the distorting effects of compression, limiting and/or microphone placement may have had a negative impact on intelligibility by reducing the effective SNR at the listener’s ears (e.g., see Cubick et al., 2018; Mansour et al., 2022).

### Challenges Associated With Conversational Sentences

So why are ECO-SiN sentences more challenging to understand than BKB sentences under similar conditions? Based on the long-term average spectra shown in the left panel of Figure 2, we may have expected the opposite result. Specifically, the increasing vocal intensity of the ECO-SiN sentences coincides with increased spectral tilt (Lu and Cooke, 2009) and a boost in mid-frequency energy relative to the BKB sentences. This frequency region is particularly relevant for understanding speech (see ANSI-S3.5., 1997) and thus could have produced a speech-intelligibility benefit for the ECO-SiN sentences that increases with increasing vocal effort. On the other hand, the right panel of Figure 2 shows that ECO-SiN sentences also contain higher modulation frequencies on average relative to BKB sentences, especially for normal and raised vocal efforts. This difference, which corresponds loosely to a faster speaking rate, may explain the increased difficulty of the ECO-SiN materials. A similar conclusion was reached by Badajoz-Davila and Buchholz (2021) who demonstrated that speech intelligibility was systematically lower when comparing the ECO-SiN sentences to BKB sentences in realistic background noise for individuals with cochlear implants. While it is known that accelerated speech interacts with speech intelligibility (Wingfield et al., 1984; Adams and Moore, 2009) if the performance difference was purely driven by speaking rate, it would be expected that intelligibility would be similar between the loud ECO-SiN vocal effort and the BKB sentences (e.g., Figure 2), however, this was not the case. There may have been additional differences between the ECO-SiN and BKB materials that are relevant here but were not explicitly analyzed, such as differences in formants or vowel space (Bradlow et al., 1996), vowel duration (Lu and Cooke, 2009), or fundamental frequency (f0) and f0 variations (Summers et al., 1988).

Another explanation for the differences in performance measured for the different speech materials in certain environments is that the complexities of the noise may have differentially interacted with the speech materials (cf. Weisser et al., 2019a, for an in-depth discussion on acoustic complexity). For example, some background noises may contain informational masking due to competing speech (e.g., advertisements are playing on a TV in the living room background noise, people are talking over a table in the dinner party background noise), which may have interfered more strongly with the conversational ECO-SiN sentences. In addition, it is well known that amplitude modulations in background noises afford individuals the ability to listen in the dips (Hopkins and Moore, 2009), and it might be that this process is more efficient for clearly spoken sentences than for natural sentences with highly unpredictable structures. It is also possible that the BKB sentences “pop-out” of the background noise more than the ECO-SiN sentences as they are incongruent with the noise in which they were presented (Hendrikse et al., 2019). Conversely, ECO-SiN sentences may blend into the realistic background noise and be harder to selectively attend. In addition, recall that the ECO-SiN sentences were also combined with reverberation that matched the realistic virtual sound environments in which they were presented. While this was done to maximize the realism of the ECO-SiN materials, adding reverberation can result in decreased speech intelligibility (Helfer and Wilber, 1990; Gordon-Salant and Fitzgibbons, 1993; Shi and Doherty, 2008).

### Limitations and Outlook

The primary reason for assessing speech intelligibility in the clinic and laboratory is to provide insight about an individual’s hearing ability in their everyday lives. However, developing more realistic speech intelligibility assessments and maintaining a level of experimental control often requires a trade-off. For example, here we used more realistic speech material from the ECO-SiN corpus and compared the sentences to BKB sentences which are typical of the materials used for speech intelligibility testing in laboratories and clinics. While the addition of realism in speech materials is a positive step for increasing realism in speech testing in order to better predict real-world performance, the sentence recall task itself is still highly unrealistic compared to how individuals communicate in the real-world. In this regard, it is important to note that many of the characteristics of natural conversational speech which are expected to benefit speech intelligibility may do so only in the full context of the task of natural conversation. For example, natural speech contains intonation that affects intelligibility (Binns and Culling, 2007; Miller et al., 2010) but also carries information such as talker emotion and cognitive state which may serve to disambiguate meaning in active conversations. It is unclear to what extent such indexical information is useful in a simple sentence repetition task with an unfamiliar talker. In real conversations, listeners can also benefit from discourse context, visual cues, shared knowledge and experience with a conversation partner, repetitions, or clarifications (Beechey et al., 2020). Accordingly, the fact that the ECO-SiN sentences were challenging to understand out of context does not mean they would necessarily be so problematic within the context of a conversation.

There is a growing body of research that aims to increase the realism of speech testing in a variety of ways (Keidser et al., 2020). For example, Best et al. (2016) evaluated a question-and-answer model based on the Helen test (Ludvigsen, 1974) which has an inherent comprehension component tapping cognitive processes used for communication in the real-world, and includes variable target talkers which mimics spatial processing required when communicating in groups in the real-world. Others have used a referential task where interactive conversations can be monitored (Beechey et al., 2019; Weisser and Buchholz, 2019). Another relevant set of studies is exploring how head orientation and movement in realistic environments intersects with speech intelligibility (Hadley et al., 2019; Hendrikse et al., 2019; Weisser et al., 2021). The inclusion of visual information in speech intelligibility testing is an area of active investigation (Devesse et al., 2020; Llorach et al., 2021) and is the next step planned for the ECO-SiN materials.

Another limitation was introduced by the applied hearing-aid platform, which mainly provided non-linear amplification and only considered an omni-directional microphone input. State-of-the-art hearing aids provide more refined implementations of compression and limiting and more advanced signal processing features, such as directional microphones and (bilateral) adaptive beamforming (e.g., Kates, 2008). Including such advanced features may have helped to overcome the negative hearing-aid benefit observed for the listeners with mild hearing loss in the louder noise environments, and potentially even provided a positive benefit. Hence, future evaluations should include state-of-the-art hearing aids to understand their benefit in the different realistic conditions and compare the results to the benefits experienced in the real world.

## Data Availability Statement

The raw data supporting the conclusions of this article will be made available by the authors, without undue reservation.

## Ethics Statement

The studies involving human participants were reviewed and approved by the Macquarie University Human Research Ethics Committee and the Australian Hearing Human Research Ethics Committee. The patients/participants provided their written informed consent to participate in this study.

## Author Contributions

KM: design and conceptualization, data curation, analysis, and writing the manuscript. TB: design and conceptualization, analysis, and writing the manuscript. VB: analysis and writing the manuscript. JB: design and conceptualization, analysis, writing the manuscript, and supervision. All authors contributed to the article and approved the submitted version.

## Conflict of Interest

The authors declare that this study received funding from Sonova AG. The funder was not involved in the study design, collection, analysis, interpretation of data, the writing of this article or the decision to submit it for publication.

## Publisher’s Note

All claims expressed in this article are solely those of the authors and do not necessarily represent those of their affiliated organizations, or those of the publisher, the editors and the reviewers. Any product that may be evaluated in this article, or claim that may be made by its manufacturer, is not guaranteed or endorsed by the publisher.

## Figures and Tables

**FIGURE A1:**
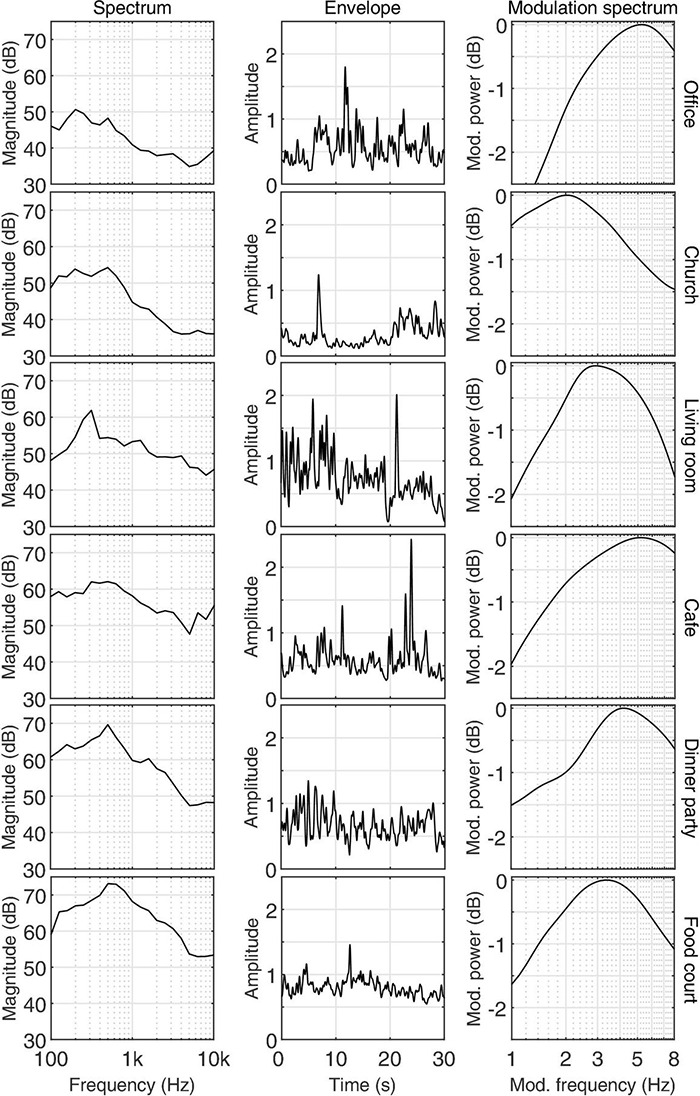
Long-term spectrum in third-octave levels (left column), temporal envelope (30 s example, center column), and modulation spectrum (right column) for the six different acoustic environments derived in free-field.

## Appendix

Figure A1 shows the long-term spectrum in third-octave levels (left column), temporal envelope (center column), and modulation spectrum (right column) for the six different acoustic environments that were derived in free-field. The spectrum and modulation spectrum were derived as described in section “Sentence Materials” considering the entire 150 s long noise signals. The temporal envelopes were derived by normalizing the noise waveforms to an RMS value of one, applying an A-weighting bandpass filter, squaring, and temporal convolution with a 0.5 s long Hann window. The figure panels show 30 s long examples of the resulting envelopes in dB.
